# Module-Based Outcome Prediction Using Breast Cancer Compendia

**DOI:** 10.1371/journal.pone.0001047

**Published:** 2007-10-17

**Authors:** Martin H. van Vliet, Christiaan N. Klijn, Lodewyk F. A. Wessels, Marcel J. T. Reinders

**Affiliations:** 1 Information and Communication Theory Group, Faculty of Electrical Engineering, Mathematics and Computer Science, Delft University of Technology, Delft, The Netherlands; 2 Bioinformatics and Statistics Group, Department of Molecular Biology, Netherlands Cancer Institute, Amsterdam, The Netherlands; 3 Mouse Models for Breast Cancer, Department of Molecular Biology, Netherlands Cancer Institute, Amsterdam, The Netherlands; National Cancer Institute at Frederick, United States of America

## Abstract

**Background:**

The availability of large collections of microarray datasets (compendia), or knowledge about grouping of genes into pathways (gene sets), is typically not exploited when training predictors of disease outcome. These can be useful since a compendium increases the number of samples, while gene sets reduce the size of the feature space. This should be favorable from a machine learning perspective and result in more robust predictors.

**Methodology:**

We extracted modules of regulated genes from gene sets, and compendia. Through supervised analysis, we constructed predictors which employ modules predictive of breast cancer outcome. To validate these predictors we applied them to independent data, from the same institution (intra-dataset), and other institutions (inter-dataset).

**Conclusions:**

We show that modules derived from single breast cancer datasets achieve better performance on the validation data compared to gene-based predictors. We also show that there is a trend in compendium specificity and predictive performance: modules derived from a single breast cancer dataset, and a breast cancer specific compendium perform better compared to those derived from a human cancer compendium. Additionally, the module-based predictor provides a much richer insight into the underlying biology. Frequently selected gene sets are associated with processes such as cell cycle, E2F regulation, DNA damage response, proteasome and glycolysis. We analyzed two modules related to cell cycle, and the OCT1 transcription factor, respectively. On an individual basis, these modules provide a significant separation in survival subgroups on the training and independent validation data.

## Introduction

Unraveling the structure of complex biological processes from genomic data sources has been a focal point in bioinformatics research. Thus far, supervised analysis of microarray data has been performed in a data-driven fashion [1]–[4]. These studies have reported and tested prognostic markers, sets of genes, which are predictive of treatment response and outcome.

One of the main issues in data-driven approaches is the small ratio of samples relative to the number of genes for a particular study, causing small sample size related problems. This problem can be addressed by reducing the number of features (input variables) or increasing the number of samples. The latter approach was pursued by combining two or even more datasets and then deriving prognostic markers from the resulting dataset [5]–[8]. Employing more samples results in, for instance, better estimates of gene variances and improves estimates of the t-statistic [9]. This approach was also followed by Segal *et al.* [10] and Tanay *et al.* [11] who constructed microarray gene expression compendia (collections of microarray data sets spanning a diversity of phenotypes).

The supervised analyses performed on compendia are data-driven and currently still employing single genes as input features. As an alternative, knowledge of functional groupings of genes into, for example pathways, can be employed to define meta-features, called modules. Such meta-features have two important advantages. Firstly, a relevant module can be directly linked to the biological processes that underly the observed outcome. Secondly, moving from a gene-based to a module-based representation reduces the number of input variables, which alleviates the small sample size problem.

Segal *et al.* [10] proposed a framework for the unsupervised knowledge-driven analysis of expression data. Within this framework, modules are extracted based on relevant gene sets from a compendium of microarray data. We follow that approach, and extend the framework to include a supervised classification analysis based on the extracted modules and the available clinical data. In addition, we introduce cancer-specific compendia, as an intermediate step between a single dataset and a complete human cancer compendium. Employing the supervised framework, we evaluate the predictive performance of classifiers derived from cancer-specific datasets, a cancer specific compendium, and a human cancer compendium. In addition, we wanted to investigate the capacity of these classifiers to generalize beyond the dataset on which they were trained. Therefore, we set up an experiment in which we validated our classifier on independent data from the same institution (intra-dataset validation), a combination of institutions (cross-dataset validation), and by validating on data from different institutions (inter-dataset validation). Finally, since we adopted the module extraction of Segal *et al.* [10], the optimized set of modules that is selected by the supervised analysis allows for a more transparent analysis of the obtained results. That is, the modules can be related to the original gene sets, and thus, to cellular processes, giving more insight into the mechanisms causing the outcome differences.

## Methods

**Figure 1:**
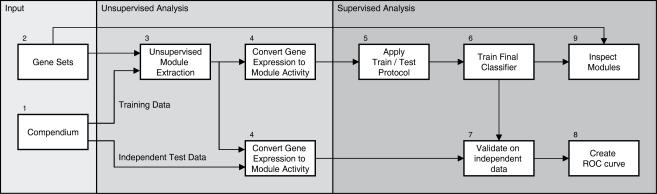
Workflow of the approach. We extended the analysis of compendia [10] to the supervised classification domain. Several microarray datasets were collected to construct compendia at various levels of underlying phenotype diversity (1). Additionally, we gathered a collection of biologically meaningful gene sets from available databases (2). Using the module extraction framework proposed by [10], we derived sets of modules (3) from these compendia and gene sets. Using these modules we construct a module activity matrix (4), allowing modules rather than single genes to be used as features. The predictive power of the different sets of modules is inspected within a classification context. Using a train/test protocol (5), we estimated the generalization error of all sets of modules [17]. Succeedingly, we trained a final classifier (6), which was then validated on independent data (7), and its performance assessed (8). Furthermore, the approach allows the final set of modules that were selected in the classifier to be compared to the original gene sets (9), allowing the identification of biological processes underlying the development and progression of cancer.

Our method extends the unsupervised knowledge-driven framework proposed by Segal *et al.* [10] to the supervised classification domain. This extension allows the identification of module-based prognostic markers, rather than gene-based markers. The entire outline of our methodology is presented in Figure 1.

### Input: Compendium

**Figure 2:**
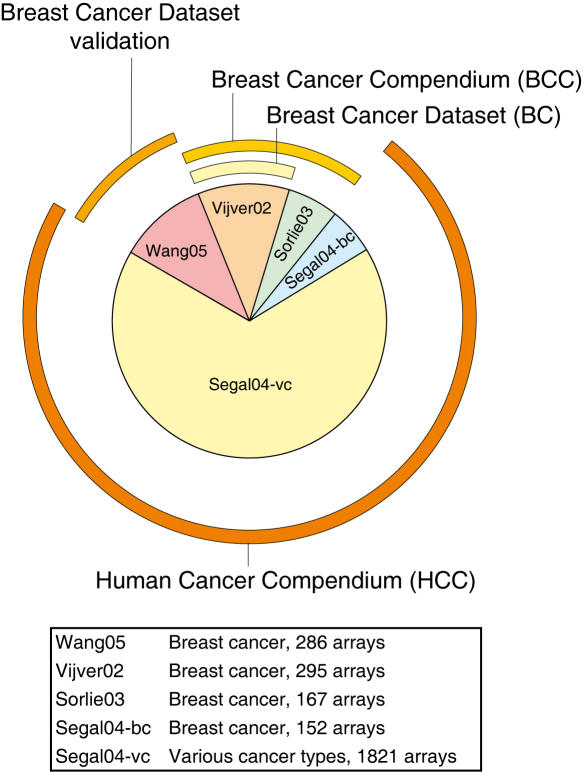
Compendia of microarray data. Microarray datasets can be grouped into compendia at various levels of underlying phenotypic diversity. The pie-chart indicates datasets from various origins, sizes, and cancer types, and the compendia are indicated by the outer rings. The ’Inter1’ training-validation configuration is depicted in this figure ([4] as training, and [3] as validation). This is one of the six configurations employed (See Table 1 for details).

The usual approach is to analyse a single microarray dataset in isolation. To find cancer related modules, Segal *et al.* [10] proposed to take multiple datasets into account that are all related to human cancer types (Figure 1, Step 1). With this global human cancer compendium (HCC), they formed modules of genes that regulate cancer in a general way. We focus on breast cancer, for which various datasets are available. We propose to construct cancer specific compendia, in our case breast cancer compendia (BCC), as an intermediate step between a single breast cancer dataset (BC) and a complete human cancer compendium (HCC), see Figure 2 and Table 1. These cancer specific compendia will reduce the small sample size problem, but at the same time should ensure coherence in underlying phenotype compared to the more global human cancer compendium. Figure 2 shows an example of how datasets from different institutions have been grouped into compendia.

**Table 1:** Experimental setup.

Features	*n*	*n_opt_*	Training	Validation	Validation
Intra/Cross-lab Validation				**Intra1**	**Cross1**
Genes	10962	48	V_1_	V_2_	V_2_+W_2_
BC (V_1_)	747	44	V_1_	V_2_	V_2_+W_2_
BCC (V_1_+W_1_+So)	911	66	V_1_	V_2_	V_2_+W_2_
HCC (Se)	1163	111	V_1_	V_2_	V_2_+W_2_
S456 (Se)	456	80	V_1_	V_2_	V_2_+W_2_
Inter-lab Validation				**Inter1**	
Genes	10962	21	V	W	
BC (V)	896	55	V	W	
BCC (V+So)	934	137	V	W	
HCC (Se)	1163	104	V	W	
S456 (Se)	456	42	V	W	
Intra/Cross-lab Validation				**Intra2**	**Cross2**
Genes	10962	101	W_1_	W_2_	V_2_+W_2_
BC (W_1_)	576	59	W_1_	W_2_	V_2_+W_2_
BCC (V_1_+W_1_+So)	911	103	W_1_	W_2_	V_2_+W_2_
HCC (Se)	1163	71	W_1_	W_2_	V_2_+W_2_
S456 (Se)	456	67	W_1_	W_2_	V_2_+W_2_
Inter-lab Validation				**Inter2**	
Genes	10962	58	W	V	
BC (W)	704	17	W	V	
BCC (W+So)	762	33	W	V	
HCC (Se)	1163	78	W	V	
S456 (Se)	456	10	W	V	

Our experimental setup allows a validation of the classifiers on data from the same institution (Intra1 and Intra2), data from the same and another institution (Cross1 and Cross2), and data from another institution (Inter1 and Inter2). In all cases the training and validation sets are non-overlapping, and thus independent. Moreover, the validation data was not used in the first step where the unsupervised approach is used to extract modules. In each of the validation schemes we included a gene-based classifier (Genes), and several module-based classifiers (BC, BCC, HCC, and S456). For each of the module-based classifiers we indicate the datasets from which the modules were extracted (Features column), along with the number of features (*n*), and the optimal number of modules/genes output from the train/test protocol (*n_opt_*). The Training column indicates the dataset on which the train/test protocol was used, and the Validation column indicates the datasets used for validation of the classifiers. All datasets are abbreviated as: V: [4], W: [3], So: [2], and Se: [10]. When we split a dataset in two equal independent parts we indicate the training (1) and validation (2) parts by subscripts.

In our analyses we have also used the HCC that was published by Segal *et al.* [10]. This compendium contains data from various cancer types and has a total of 1973 arrays, for which 14143 genes are present. The compendium already contained data from three previous breast cancer studies, in total 152 arrays: 26 arrays from the first study by Perou *et al.* [12], 41 arrays from the second study by Perou *et al.* [13], and 85 arrays from Sorlie *et al.* [14].

The additional breast cancer microarray datasets that we have used, originate from previously published research [2]–[4]. The Vijver dataset consists of 295 breast cancer patients, the Wang dataset consists of 285 records, and the Sorlie data consists of 167 records. To be able to use these datasets in conjunction with the HCC, we mapped all the available probes to the same set of Entrez ids in the HCC. Furthermore, after mean-normalisation, we discretized each dataset separately into three levels: induced (1), basal (0), and repressed (−1), taking into account the skewing and variance in each of the datasets (Discretization was applied, because the module extraction procedure that Segal *et al.* [10] proposed, requires a discrete input.).

Outcome data (time to metastasis) was available for all patients in the Vijver and Wang datasets. For classification, the poor outcome group was defined as all patients with time to metastasis less than five years, and the good outcome group as those with time to metastasis greater than or equal to five years. Censored patients in the poor group were not taken into account when training and assessing a classifier. On the other hand, censored patients in the good group were included in both the training and validation [15].

### Input: Gene Set Collection

**Figure 3:**
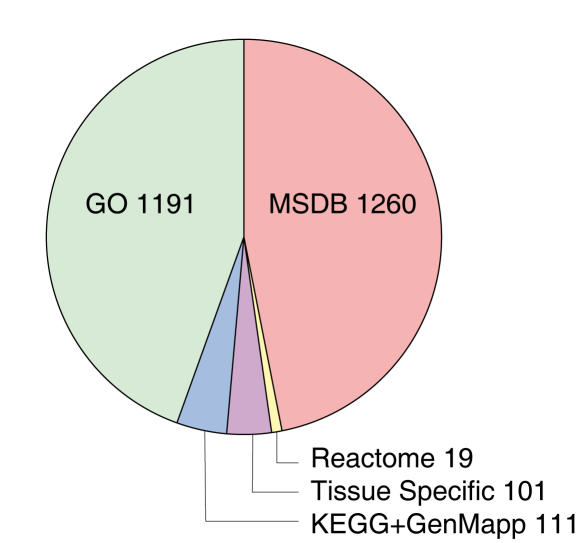
Pie chart indicating the origin of the gene sets. A total of 2682 gene sets were collected. The GO, KEGG, GenMapp, and Tissue specific gene sets were taken from the study by Segal et al. [10]. The Reactome pathways were downloaded from the Reactome website [23], and the MSDB gene sets were taken from the molecular signature database [24].

We collected 2682 gene sets from several biological databases and resources (Figure 1, Step 2), including some additional databases that were not included in the collection of gene sets used by Segal *et al.* [10], see Figure 3. In the original analysis presented by Segal *et al.* [10], approximately half of the gene sets were obtained by performing hierarchical clustering on the expression data. We chose to omit any hierarchical clusters in the collection of gene sets, as the inclusion of hierarchical clusters would introduce an additional data-driven bias. As a result, our analysis is more knowledge-driven when compared to the original study.

### Unsupervised Analysis

To extract modules from compendia of microarrays, we largely followed the knowledge-driven approach proposed by Segal *et al.* [10] (Figure 1, Step 3). In short, this unsupervised method finds modules in (compendia of) discretized microarray data. A module is defined as a subset of genes with correlated expression across a set of arrays, and is constructed by combining (parts of) gene sets based on discretized gene expression data. The module extraction process can be seeded by biologically relevant gene sets (extracted from e.g. GO and KEGG), thus incorporating a knowledge-driven component in the analysis. An in depth description of the procedure is given in the supplementary Text S1, and supplementary Figure S1.

Following the extraction of the modules, a module activity matrix is constructed for the training data as well as the validation data (Figure 1, Step 4). The module activity matrix represents the behavior of the group of genes in a module by a discrete variable.

The conversion from gene expression to module activity is done per array, per module. For the induced and repressed genes separately, we test whether the overlap of induced or repressed genes on the array with the module is significant, compared to a random draw. To this end, we use the hypergeometric distribution to calculate a p-value for the significance of the overlap. Following FDR correction [16] (significance threshold = 0.05) one of the following four situations occur:

- Neither p-value is significant: the module activity is basal (i.e. 0)
- Only the induction p-value is significant: the module is induced (i.e. 1)
- Only the repression p-value is significant: the module is repressed (i.e. −1)
- Both p-values are significant: the module activity is basal (i.e. 0)

**Figure 4:**
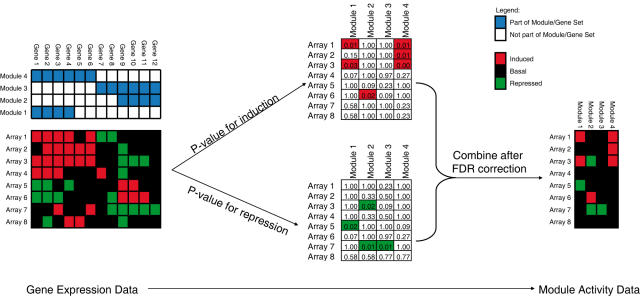
Converting gene expression data into module activity data. For a given gene expression dataset, and a set of modules we assessed the statistical significance of the overlap of induced/repressed genes with the modules using the hypergeometric distribution. This leads to two p-values for each array/module pair. These p-values are combined into a single discrete module activity score.

Figure 4 presents an example of a set of microarrays and modules that are converted to a module activity matrix.

For each of the validation schemes (Table 1) we constructed several module-based classifiers (BC, BCC, HCC, S456) based on modules extracted from the datasets indicated in the Features column of Table 1.

### Supervised Analysis

Supervised classification provides a means to identify modules with activities that are significantly associated with some relevant outcome variable, such as, metastasis-free survival in breast cancer (Figure 1, Step 5). To obtain an unbiased estimate of the generalization error of the different sets of modules, we used a double-loop cross validation procedure [17].

In our experiment, we focused on differences between the sets of modules and we omitted an extensive evaluation of a range of different classifier types. Since all features are discrete, we used forward filtering as module selector, the mutual information as criterion to evaluate the individual modules (using maximally 200 modules), and a simple Bayes classifier [18]. For the discretized gene-expression data we used the same setup as for the module-based approach.

Following the Train/Test procedure, we trained a final classifier (Figure 1, Step 6). This classifier was trained using the top ranked features, that were estimated in the train/test protocol. The final classifier was validated on an independent dataset (Figure 1, Step 7), which had not been employed in any of the training steps (Figure 1, Steps 2–6).

To assess the performance of the classifiers on the independent validation dataset, we constructed an ROC curve (Figure 1, Step 8). To compare the performance of various feature types we adopted the area under the curve (AUC) as a performance measure.

Finally, a feedback step relates the modules selected in the classifier to the original gene sets (Figure 1, Step 9). This allows direct insight into the underlying mechanisms, compared to the annotation lookup of single genes in terms of functional groups in data-driven approaches.

### Experimental Setup

We wanted to investigate the capacity of our classifiers to generalize beyond the dataset that they were trained on. Therefore, we designed our experiments such that three different validation schemes were possible. In all cases the training and validation sets are non-overlapping (independent), i.e. no samples that were used during module extraction/training are employed in the validation. The following three scenarios were considered: training and validation on data from 1) the same institution (denoted as intra-lab validation); 2) a combination of the same and other institutions (cross-lab validation); and 3) separate institutions (inter-lab validation). Since we had equivalent outcome data for the Vijver and Wang datasets, we mirrored the role of both so that we ended up with a total of six experiments, as shown in Table 1.

## Results and Discussion

### Extracting modules from the compendia

For each of the compendia we derived a set of modules using the discretized gene expression data as well as the 2682 gene sets as input. The number of modules that were found are listed in Table 1. The number of modules found ranged from 576 to 1163, which is a significant reduction in the number of features from the original 14143 genes. Additionally, we included the previously published 456 modules [10] in the current investigation (S456). These differ from the HCC modules, as they are constructed based on gene sets derived by hierarchical clustering.

### Classification performances

**Figure 5:**
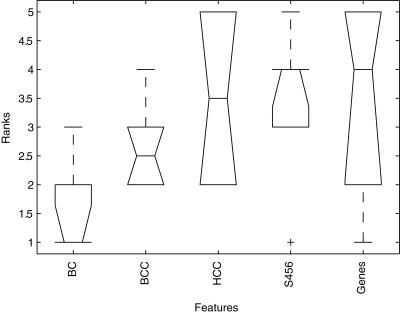
Boxplot showing ranked AUC results. Boxplot showing the median ranks of the performance of each of the five feature types across the six experiments (see Table 1). In each of the six experiments the features were ranked based on the AUC obtained on the independent validation set (1 best, 5 worst).

The classification performances of the experiments listed in Table 1, are compared based on the AUCs obtained on the validation data. For each of the six experiments (Intra1, Cross1, Inter1, Intra2, Cross2, Inter2) the results obtained with each feature type (BC, BCC, HCC, S456, Genes) were ranked based on the AUC. Figure 5 shows a boxplot of the ranks obtained for each of the feature types. A table containing all individual AUC values and ranks is presented in the supplementary information (Table S1). The median rank of the BC features is the lowest of all the feature sets, the BCC median rank is slightly worse, but still better than the HCC, S456, and Genes features which perform the worst.

**Figure 6:**
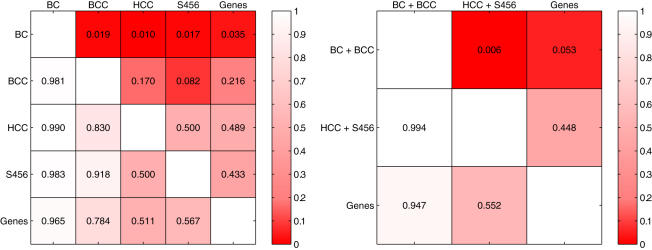
Pairwise comparison of the five feature types. Each cell (row = i, column = j) depicts the the p-value obtained by performing a one-sided Wilcoxon rank sum test with as alternative hypothesis that the median rank of type i is lower than type j, based on the AUCs achieved for each of the six experiments. The plot on the left shows individual comparisons, the plot on the right includes comparisons of groups of features. Cell-shading reflects the p-values.

To assess the statistical significance of the observed differences between the median ranks, we applied a one-sided Wilcoxon rank sum test to all pair-wise combinations of feature types. The obtained p-values are depicted in the left panel in Figure 6. We also employed the Wilcoxon rank sum test to perform pair-wise comparisons between the feature types derived from breast cancer compendia (BC+BCC), features types derived from human cancer compendia (HCC+S456) and Genes. The results are depicted in the right panel of Figure 6.

From the left panel in Figure 6 we can conclude that, although the BCC modules have a lower median rank compared to the HCC modules, S456 modules, and Genes, there is not enough statistical evidence (at the 0.05 level) to support the claim that the BCC modules outperform the HCC modules, S456 modules, or the Genes. Since the BCC modules are derived from a larger collection of data than the BC modules, we would have expected a performance that is at least equal and possibly even better than the BC modules. Since breast cancer is known to be a heterogeneous disease, we hypothesize that differences in the subtype composition of the datasets cause the poorer performance of the BCC modules.

From the right panel in Figure 6, we can, however, conclude that the BC and BCC modules jointly perform better than the HCC, and S456 modules (p = 0.006). This indicates that a human cancer compendium lacks specificity with respect to a breast cancer compendium. We can therefore conclude that for breast cancer specific prediction, a cancer specific compendium is more suitable compared to a more global human cancer compendium. As shown by Segal *et al.* [10] the HCC and S456 modules may still be relevant for identifying differences between cancer types.

The pairwise comparisons (left panel in Figure 6) indicate that the median rank of the BC modules is better than each of the other feature types (all p<0.05). Moreover, gene-based classifiers show a very large variability in comparison to BC module-based classifiers (see Figure 5). One possible explanation for this observation is that the conversion of gene expression data to module activity data may, in fact, function as a form of regularization which removes noise. Typically, the amount of regularization needs to be optimized for a given classifier. We hypothesize that the fact that genes in a module are roughly associated with the same biological process, ensures an optimal degree of regularization.

For a given classifier to serve as a prognostic index in clinical practice, a suitable operating point on its associated ROC curve needs to be selected. For outcome prediction in breast cancer, the True Positive Rate (TPR) is typically set at a desired threshold, and based on the ROC curve, the corresponding False Positive Rate (FPR) possible is determined. Therefore, we have re-evaluated the AUC scores by integrating over the TPR interval ranging from 0.5 to 1. This interval was chosen since it reflects a clinically more relevant range than the complete TPR range ([0,1]). All results are reported in the supplementary information (Table S2, Figure S2 and Figure S3). Consistent with earlier results, the BC modules perform significantly better compared to all other feature types. In addition, the BCC modules now have a significantly lower median rank compared to the HCC and S456 modules (p = 0.05, and p = 0.01). This strengthens our conclusion that the BC modules perform better than the other feature types, and that a breast cancer specific compendium performs better compared to a human cancer compendium, especially when considering a clinically relevant setting.

### Interpretability of Gene and Module-based signatures

All classifiers output a signature of relevant features, that is predictive for survival. To better understand the biological processes associated with disease outcome in breast cancer, the signatures are further investigated. For gene-based features, the overlap with known pathways is employed to attach biological meaning to an obtained signature. Module-based classifiers, on the other hand, return a set of predictive modules rather than single genes. Each of these individual modules may provide links to relevant biological processes. Moreover, since modules are extracted from the data by combining (parts of) gene sets, these may link additional genes to known pathways, which are relevant to disease progression in breast cancer.

To explore the association of the signatures to biological processes, we analyzed a gene-based and BC module-based signature. We chose to compare the gene-based signature and BC module-based signature from Inter1 (Table 1), since they were derived from the same dataset. These signatures consist of 21 genes, and 55 modules (Dataset S1), respectively.

For every module in the module-based signature, as well as the 21 single-gene signature, we computed the enrichment for each of the 2682 gene sets employing the hypergeometric distribution. For the gene-based signature no gene sets were significantly enriched (*p*<0.05 after Bonferroni correction), whereas 319 gene sets were significantly enriched in at least 1 of the 55 modules in the module-based signature (*p*<0.05 after Bonferroni correction). The complete matrices of raw p-values are depicted in supplementary information (Text S1, and Figure S4).

**Figure 7:**
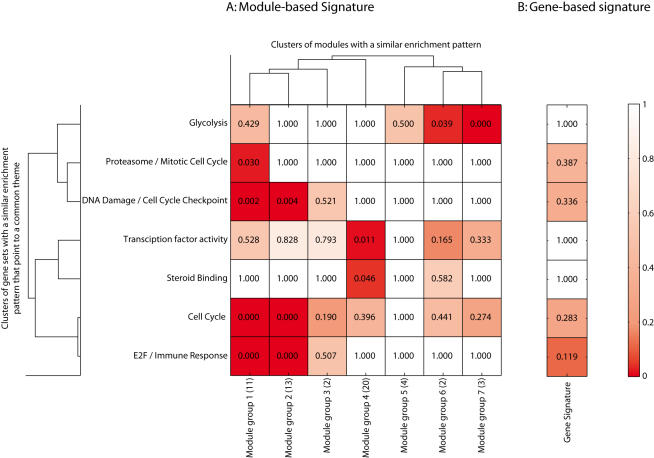
Comparison of a module-based signature (A) and a gene-based signature (B). The module-based signature from the Inter1 experiment contains 55 modules, and the gene-based signature contains 21 genes (Table 1). For both signatures an enrichment score for their overlap with the collection of 2682 gene sets was calculated based on the hypergeometric distribution. This resulted in a total of 319 gene sets that were enriched in at least one module or in the gene-based signature (*p*<0.05 after Bonferroni correction), see supplemental figure S4. Several modules turned out to have a similar pattern of enrichment across the gene sets. Additionally, gene sets that relate to a common theme turned out to have a similar enrichment pattern across the modules. Therefore, we clustered the matrix of p-values in both dimensions (2-dimensional, hierarchical clustering, complete linkage, Euclidean distance). The dendrograms at the top, and to the left indicate the clustering, where we chose to group either dimension into seven distinct groups. The labels on the left indicate the most common biological theme, and the label on the bottom indicates the groups of modules formed along with the number of modules in each group in brackets. The main table shows the median p-value for the enrichment of each of the seven clusters of modules, across these seven groups of gene sets. Similarly, the table on the right shows the median p-values for the gene signature. Shading of the cells reflects the p-values.

Many of the 319 gene sets are associated with similar biological processes within the context of the 55 module signature (i.e. they have similar enrichment profiles across the 55 modules). Therefore, we clustered the gene sets based on enrichment scores into seven distinct clusters employing complete linkage, hierarchical clustering with Euclidean distance as dissimilarity measure. The common biological themes associated with the gene sets in each of the resulting seven clusters are listed on the left in Figure 7.

Based on the complete table of enrichment p-values (Supplementary Figure S4), it is evident that there are clusters of modules that also show a similar enrichment pattern across the gene sets. Therefore, we clustered the modules into seven distinct groups, as depicted by the dendrogram at the top in supplementary Figure S4. These clusters were labeled Module groups 1 to 7, as indicated at the bottom in Figure 7.

The main table in Figure 7 shows the aggregated enrichment p-values. More specifically, cell (*i*, *j*) depicts the median enrichment p-value for all modules in module group *j* with respect to all gene sets in gene set group *i*. The column vector on the right shows the median enrichment scores for the groups of gene sets with respect to the single gene signature.

Figure 7 shows several strong links of the module-based signature to biological processes. Five out of the seven groups of modules can be linked to biological processes that are known to be involved in cancer progression (Cell Cycle, DNA Damage, E2F transcription factors, and Proteasome). Most of these have been previously related to breast cancer [19]. It is interesting to note that glycolysis has only recently been identified as a key factor in tumor progression [20].

### A detailed analysis of two modules

**Figure 8:**
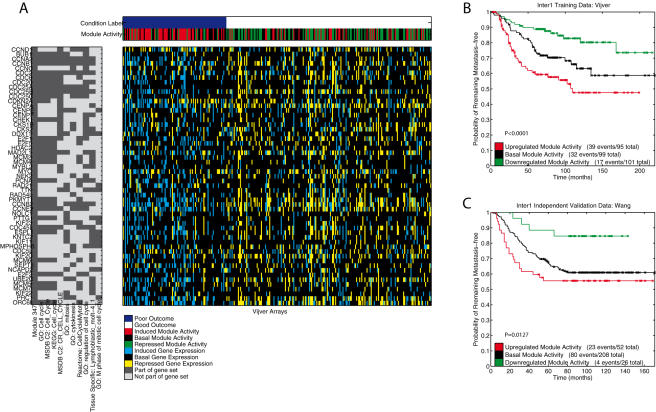
A cell cycle related module. A) Module activity data of a Cell Cycle related module (Module group 2 in Figure 7) that was extracted from the Vijver [21] data (Inter1, Table 1). The top heatmap shows the binary condition label, and the discrete module activity data (rows), for all the Vijver arrays (columns) [4]. Arrays are ordered according to the metastasis free survival time. The heatmap in the middle shows the discrete gene expression data for the 55 genes (rows) in the module. On the left, a binary heatmap shows the 55 genes, along with the gene sets that show the most significant overlap with this module. The gene sets are ranked based on their p-value for the overlap with the module (hypergeometric distribution), we show the top 10 gene sets (p-values ranging from 10^−51^ to 10^−25^, all significant at *p*<0.05 after Bonferroni correction). On the right, two Kaplan-Meier curves indicate the predictive power of this module when arrays with the same module activity are grouped. B) The Kaplan-Meier curves for the three groups defined by the activity of this module on the Vijver [21] data (Inter1 training, Table 1). C) The Kaplan-Meier curves for the three groups defined by the activity of this module on the independent [3] data (Inter1 test data, Table 1). The legend indicates the three groups and lists the number of events and total number within the groups. P-values correspond to the logrank test.

**Figure 9:**
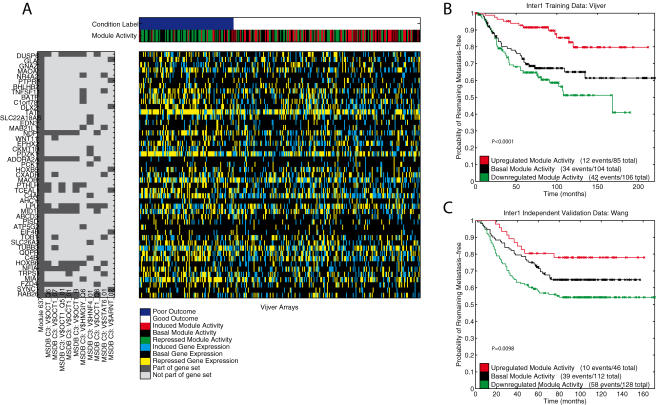
An Oct1 related module. A) Module activity data of an OCT1 transcription factor related module (Module group 4 in Figure 7) that was extracted from the Vijver [21] data (Inter1, Table 1). The top heatmap shows the binary condition label, and the discrete module activity data (rows), for all the Vijver arrays (columns) [4]. Arrays are ordered according to the metastasis free survival time. The heatmap in the middle shows the discrete gene expression data for the 47 genes (rows) in the module. On the left, a binary heatmap shows the 47 genes, along with the gene sets that show the most significant overlap with this module. The gene sets are ranked based on their p-value for the overlap with the module (hypergeometric distribution), we show the top 10 gene sets (p-values ranging from 10^−13^ to 10^−7^, all significant at *p*<0.05 after Bonferroni correction). On the right, two Kaplan-Meier curves indicate the predictive power of this module when arrays with the same module activity are grouped. B) The Kaplan-Meier curves for the three groups defined by the activity of this module on the Vijver [21] data (Inter1 training, Table 1). C) The Kaplan-Meier curves for the three groups defined by the activity of this module on the independent [3] data (Inter1 test data, Table 1). The legend indicates the three groups and lists the number of events and total number within the groups. P-values correspond to the logrank test.

Based on the module activity representation, the arrays in a dataset can be separated into 3 groups: arrays where the module is activated, repressed or showing basal activity. Using this separation, we present the Kaplan-Meier curves for two modules from the module-based signature, on the training data [4], as well as on the independent validation data [3], see Figures 8 and 9.

Figure 8 shows a module (from Module group 2) that has a highly significant overlap with Cell Cycle related gene sets (enrichment of the top gene set: *p*<10^−51^), see Figure 7. This module is significantly associated with disease progression on both the datasets from Vijver *et al.* [4] (*p<*0.0001) and Wang *et al.* [3] (*p* = 0.0127). Deregulation of the cell cycle has been identified as one of the hallmarks of cancer [19]. More importantly, an increased activity of the cell cycle has been linked to more aggressive tumors. This is in accordance with our observation for this module, which shows that an induced module activity is linked to the subgroup with the worst outcome. Conversely, a repressed module activity shows the best outcome.

Figure 9 shows a module (from Module group 4) that has a significant enrichment for OCT1 transcription factor related gene sets (enrichment of the top gene set: *p*<10^−12^). The Kaplan-Meier curves show a significant separation between the induced, basal, and repressed module activities on both the Vijver *et al.* [4] data (*p*<0.0001), and the Wang *et al.* [3] data (*p* = 0.0098). In breast cancer, the OCT1 transcription factor is known to be often overexpressed [21] relative to normal breast tissue, but its exact role in the tumorigenic process has remained unclear. Additionally, OCT1 has been identified as a transcriptional repressor [22]. We show that the concerted repression of downstream targets of the OCT1 transcription factor relates to a poor outcome group. On the other hand, an induced module activity relates to a subgroup with significantly better outcome. Thus, this module can be identified as a potential tumor suppressor module.

### Conclusion

By extending an existing unsupervised knowledge-driven framework to the supervised classification domain, we were able to investigate the effects of including knowledge from previous gene expression studies (through compendia) as well as known cellular processes (through gene sets) on the accuracy of outcome prediction in breast cancer. Our analysis included a validation of the classifiers on independent data, which allowed for an objective evaluation of the actual generalization behavior of the gene-based and module-based classifiers in a clinically relevant setting.

Classifiers based on genes had a very large variance, compared to the BC module-based classifier. We hypothesize that the conversion of gene expression data to module activity functions as a regularization step, where the extent of the regularization is controlled by the specificity of the modules, resulting in more stable classifiers.

Overall, a trend emerges in the performance versus compendium specificity. Modules from the most specific single dataset showed the best performance-significantly outperforming all other classifiers. These were closely followed by modules extracted from a breast cancer specific compendium, which performed significantly better than modules from the human cancer compendium when evaluated across a clinically relevant TPR range. Finally, modules from the human cancer compendium showed the weakest performance. This indicates that it is preferable to employ a compendium specific to the cancer type under study. Moreover, the heterogeneity between different institutions tends to be more detrimental than the gain in sample size when a breast cancer specific compendium is constructed.

A module-based approach to classification provides a signature of predictive modules, as opposed to a gene-based signature. Interpretation of a gene-based signature is usually limited to a mapping of the genes in the signature to functional categories. However, for the approach outlined here, it holds that the modules were constructed from biologically meaningful gene sets, and therefore these can be linked directly to the underlying biological processes. We illustrated this advantage by providing a meta-representation of the modules in one of the module-based classifiers, which reveals molecular processes, such as cell cycle, DNA damage, glycolysis, and proteasome, known to be involved in breast cancer. The gene-based signature provided no significant links at all. This gain in biological insight greatly favors the use of a module-based classifier.

Our research includes an in-depth analysis of two modules that were part of the module-based signature, which were related to cell cycle, and the OCT1 transciption factor. By themselves, these modules provide a significant separation in subgroups on the training and independent validation data. The cell cycle related module indicated that an induced module activity is linked to the worst outcome. This confirms the well known relationship between the cell cycle process and cancer in general. On the other hand, the OCT1 related module revealed a novel relationship to breast cancer outcome. Based on its module activity, this module could be designated as a tumor repressor module. Neither of these factors could be revealed from the gene-based signature. Therefore, we conclude that module-based signatures provide a much richer insight to the underlying biology compared to gene-based signatures.

Research on outcome prediction not only contributes to the development of reliable diagnostic tests, but also by improving our understanding of the processes involved in carcinogenesis, and specifically how these influence disease progression and therapy response. From a practical perspective, diagnostic tests based on small gene sets are preferred, and are also designed with this objective in mind. However, such sets often fail to provide significant biological insight into the disease. Our module-based classifiers were not designed to employ a minimal number of genes, and the large number of genes employed could be a limitation to the direct application of these classifiers in a clinical setting. However, in our study, the module-based classifier had a significantly lower variance in performance than the gene based classifier, a property which is clearly preferable in the clinical setting. We clearly demonstrate that the module-based gene sets provide a much richer feedback by revealing functional categories associated with disease outcome. These insights could speed up the development of anti-cancer drugs, since the identified processes will help focus the search for viable drug targets. In conclusion, while module-based classifiers are perhaps less practical for clinical use due to the large gene sets being employed, their robustness and the biological insights they provide will most likely result in both short and long term clinical benefit.

## Supporting Information

Text S1 Module-based outcome prediction using breast cancer compendia.
(0.11 MB DOC)

Figure S1 Methodology overview. Overview of the unsupervised module extraction procedure, followed by a supervised investigation of the relation between module expression and conditions. In this example no FDR correction was done, so as to retain a fair amount of significantly expressed gene sets/modules.
(1.16 MB EPS)

Figure S2 Boxplot showing ranked AAC results. In each of the six experiments the features were ranked based on the AAC (TPR range from 0.5 to 1) obtained on the independent validation set (1 best, 5 worst). This boxplot shows the median rank along with the quartile ranges for each of the five features.
(0.59 MB EPS)

Figure S3 Comparison of ranked AAC results. Two tables showing a pairwise comparison of the five feature types. Each cell (row = i, column = j) depicts the the p-value obtained by performing a one-sided Wilcoxon rank sum test with as null hypothesis that the median rank of type i is lower than type j, based on the AACs (TPR range from 0.5 to 1) achieved for each of the six experiments. The plot on the left shows individual comparisons, the plot on the right includes comparisons of groups of features. Cell-shading reflects the p-values.
(0.81 MB EPS)

Figure S4 Comparison of a module-based signature (A) and a gene-based signature (B). The module-based signature from the Inter1 experiment contains 55 modules, and the gene-based signature contains 21 genes (Table 1). For both signatures an enrichment score for their overlap with the collection of 2682 gene sets was calculated based on the hypergeometric distribution. This resulted in a total of 319 gene sets that were enriched in at least one module or in the gene-based signature (P<0.05 after Bonferroni correction). Several modules turned out to have a similar pattern of enrichment across the gene sets. Additionally, gene sets that relate to a common theme turned out to have a similar enrichment pattern across the modules. Therefore, we clustered the matrix of p-values in both dimensions (2-dimensional, hierarchical clustering, complete linkage, Euclidean distance). The dendrograms at the top, and to the left indicate the clustering, where we chose to group either dimension into seven distinct groups. The labels on the right indicate the individual gene set labels, and the label on the bottom indicates the groups of modules formed along with the number of modules in each group in brackets. The main table shows the median p-value for the enrichment of each of the seven clusters of modules, across these seven groups of gene sets. Similarly, the table on the right shows the median p-values for the gene signature. Shading of the cells reflects the p-values.
(4.12 MB EPS)

Dataset S1 (5.69 MB XLS)

Table S1 (0.03 MB DOC)

Table S2 (0.03 MB DOC)
