# Supplementary material for: Module-Based Outcome Prediction Using Breast Cancer Compendia
Source: PLoS One. 2007 Oct 17;2(10):e1047. doi: 10.1371/journal.pone.0001047 (PMC2002511; doi:10.1371/journal.pone.0001047)
Supplement: Text S1 — Module-based outcome prediction using breast cancer compendia. (0.11 MB DOC) [file pone.0001047.s001.doc]

Module-based outcome prediction using breast cancer compendia:

Supplementary text

Van Vliet, Martin H. (M.H.vanVliet@TUDelft.nl), and Klijn Christiaan N., and Wessels, Lodewyk F.A., and Reinders, Marcel J.T.

Information and Communication Theory Group, Faculty of Electrical Engineering, Mathematics and Computer Science, Delft University of Technology, Mekelweg 4, 2628 CD Delft, The Netherlands

Bioinformatics and Statistics group, Department of Molecular Biology, Netherlands Cancer Institute, Plesmanlaan 121, 1066 CX Amsterdam, The Netherlands

Mouse models for breast cancer, Department of Molecular Biology, Netherlands Cancer Institute, Plesmanlaan 121, 1066 CX Amsterdam, The Netherlands

# Supplementary Information

The general consensus in clustering is the existence of groups of genes that exhibit a similar behavior for all arrays [1]. However, this is rather restrictive when investigating compendia of data. In this case, we would like to find statistically significant patterns in subsets of genes and arrays. Especially when compendia contain a large diversity of phenotypes, this is the case.

Finding modules in a compendium of microarray data is in fact equivalent to the problem of discovering bi-clusters. This mathematically challenging task has been proven to be NP-Hard [2]. For example, a supervised learning algorithm [3], a node-deletion algorithm [4], coupled two-way clustering [5], a signature method [6], and an approach based on Gibbs sampling [7], have been proposed to find bi-clusters. All bi-clustering methods are capable of finding a single bi-cluster in the data based on an optimality criterion. However, all methods share the problem that finding several, possibly overlapping, bi-clusters is difficult. In general a form of masking of already discovered bi-clusters is applied to prevent the algorithm from repetitively finding the same bi-cluster. These masking procedures in fact limit the ability of the algorithm to discover overlapping bi-clusters in the data. The recently published module extraction algorithm [8] in fact exploits the masking procedure by using biological knowledge to provide a masking in the gene-dimension. The proposed framework uses gene sets, originating from GO, KEGG pathways, GenMapp pathways, and hierarchical clusters, to guide the search for bi-clusters. A module is formed by combining several gene sets, and allowing non-consistently behaving genes to be thrown out. The key concept is that the pattern of over-representation of induced/repressed genes in a module is only observed for a subset of the arrays. A more in depth description is provided in the Module extraction procedure section.

# Dataset Integration

The compendia of microarray data contain datasets from different platforms. Prior to discretization and combining the datasets, we normalized the affymetrix type of datasets. All intensity values lower than 10 were set to 10. The actual normalisation followed this rule [8]:

$$g_{i,j}\to log(g_{i,j})-log(\overline{g_{j}})=log(\frac{g_{i,j}}{\overline{g_{j}}})$$

$\forall i=1:nArrays j=1:nGenes$ (1)

where $g_{i,j}$ is the intensity on a particular array $i$ of a gene $j$, and $\overline{g_{j}}$ is the mean intensity of gene $g_{j}$ over all arrays. This normalization mimics a 2 channel microarray where the reference channel is a pool of all samples under consideration.

# Module Extraction

To extract modules from microarray data, and more specifically from compendia, Segal et al. [8] introduced an approach based on evaluation of the statistical significance of over-/under-representation of gene sets. In analogy to the method proposed by Segal we used the following step-by-step approach (see also Figure S1):

- Step 1: Evaluate the statistical significance of all array-gene set pairs by evaluating the hypergeometric distribution. For each array-gene set pair, this results in two p-values, one for over-representation of induced, and one for repressed genes in the gene set. This results in the discrete Arrays-vs-Gene sets matrix.
- Step 2: For all significant arrays-gene set pairs the average number of induced (or repressed) genes is used. This data is clustered in the gene set dimension (Hierarchical, Pearson correlation, average linkage).
- Step 3: Each node in the dendrogram with a difference in pearson correlation to its parent node $\geq0.05$ is considered a module. The module is formed by taking the union of the clustered gene sets.
- Step 4: Similar to step 1, using the hypergeometric all array-module pairs are evaluated, thus forming the module expression data. An Arrays-vs-Modules matrix is formed.
- Step 5: For each gene in each module an evaluation is made whether the expression of the gene is consistent with the module expression. Inconsistent genes are then removed from the module. We used the same method as [8].
- Step 6: Similar to step 1, a final Arrays-vs-Modules matrix is formed for the set of modules: the module activity matrix
- Step 7: Finally, a Modules-vs-Conditions map is formed by evaluating the statistical significance of each Module-Condition pair. More specifically, the hypergeometric distribution is employed to assess the significance in overlap between a module being active or repressed and a particular condition variable.

At all steps in the module extraction procedure significance thresholds were set at 0.05, and multiple testing was taken into account by FDR correction [9].

The map obtained in the final step of the procedure allows a primary inspection of possible relations between module expression and clinical properties. In addition to this map, we propose to extend the map by also evaluating the significance of modules versus the complement conditions (see Figure S1).

In the original paper, [8] reported that their analysis of the HCC data revealed a total of 456 modules with a significant relation with one or more of the arrays in the compendium. In the current investigation these modules (S456) were also included.

Matlab scripts are available upon request.

# Classification Method

Wessels et al. [11] described a generally applicable framework for building diagnostic classifiers from high throughput data. We adopted this methodology combined with forward filtering as feature selector, and the mutual information as criterion to evaluate the individual features (using maximally 200 modules), and a simple Bayes classifier [10] for the module activity data. The training and validation procedure was performed employing 10 repeats of 10 fold cross validation in both the inner (training) and outer (validation) loop. At all points data splits were stratified with respect to the class prior probabilities.

The method can be described in a few steps:

1. Firstly, the data is split in a train and validate part, the repeat.
2. Next, the train data is split again in a train_train and train_test dataset.
3. On the train_train part a 10-fold cross validation is performed to estimate the optimal number of features $n*$.
4. A final classifier is trained on the train data, using the top $\overline{n*}$ ranked features, where $\overline{n*}$ is the average of the 10 $n*$.
5. Finally, the classifier performance is assessed on the validation set.

In the inner loop (step 4), learning curves are constructed based on the average false positive false negative ratio, which is defined as:

(2)

where $e_{FPFN}$ represents the average false positive false negative ratio error, $TP$ the number of true positives, and $TN$ the number of true negatives. In each iteration in the inner loop the $n*$ is defined as the number of features at which the $e_{FPFN}$ is minimal.

The classifiers were compared to each other based on the ROC curves for each of them on the validation dataset. Matlab scripts are available upon request.

# Supplementary Results: Classification performance

In each of the six experiments we created ROC curves for each feature type. Table 1 shows the obtained AUC values, along with the ranking of the different feature types.

Additionally, we repeated the ranking of all methods with the area above the curve (AAC) calculated over the TPR interval ranging from 0.5 to 1. This is an interval which is clinically more relevant, since this typically involves setting the TPR to a certain threshold and using the corresponding best possible FPR. Table 2 lists the AAC values obtained. The median ranks observed for the features was visualized in a boxplot, Figure S2. Lastly, we also compared the different feature sets by applying the Wilcoxon rank sum test for equal medians, Figure S3

# Supplementary Results: Interpretability of Gene and Module based signatures

A full table showing all individual module-gene set enrichment scores is presented in Figure S4. The 55 modules are supplied as supplementary Dataset S1.

# References

[1] Eisen,M., Spellman,P., Brown,P. and Botstein,D. (1998) Cluster analysis and display of genome-wide expression patterns. *PNAS,* 95: 14863–8.

[2] Johnson,D. (1987) The NP-completeness column: an ongoing guide. *J. Algorithms,* 8: 438–88.

[3] Califano,A., Stolovitzky,G. and Tu,Y. (2000) Analysis of gene expression microarrays for phenotype classification. In *Proc Int Conf Intell Syst Mol Biol.*

[4] Cheng,Y. and Church,G. (2000) Biclustering of expression data. In *Proc Int Conf Intell Syst Mol Biol.* 8: 93–103.

[5] Getz,G., Levine,E. and Domany,E. (2000) Coupled two-way clustering analysis of gene microarray data. *PNAS,* 97: 12079–12084.

[6] Ihmels,J., Friedlander,G., Bergmann,S., Sarig,O., Ziv,Y. *et al.* (2002) Revealing modular organization in the yeast transcriptional network. *Nat. Genet.,* 31: 370–377.

[7] Sheng,Q., Moreau,Y. and de Moor,B. (2003) Biclustering microarray data by gibbs sampling. *Bioinformatics,*  19: ii196–ii205.

[8] Segal,E., Friedman,N., Koller,D. and Regev,D. (2004) A module map showing conditional activity of expression modules in cancer. *Nat. Genet.,* 36: 1090–8.

[9] Benjamini,Y. and Hochberg,Y. (1995) Controlling the false discovery rate: a practical and powerful approach to multiple testing. *Journal of the Royal Statistical Society, Series B,* 57: 289–300.

[10] Domingos,P. and Pazzani,M. (1996) Beyond independence: conditions for the optimality of the simple bayesian classifier. In *ICML*.

[11] Wessels,L., Reinders,M., Hart,A., Veenman,C., Dai,H., *et al.* (2005) A protocol for building and evaluating predictors of disease state based on microarray data. *Bioinformatics,* 21: 3755–62.

[12] Van de Vijver,M., He,Y., van ’t Veer,L., Dai,H., Hart,A., *et al.* (2002) A gene-expression signature as a predictor of survival in breast cancer. *N. Engl. J. Med.,* 347: 1999–2009.

**Figure S1. Methodology overview.**

Overview of the unsupervised module extraction procedure, followed by a supervised investigation of the relation between module expression and conditions. In this example no FDR correction was done, so as to retain a fair amount of significantly expressed gene sets/modules.

**Figure S2. Boxplot showing ranked AAC results**

In each of the six experiments the features were ranked based on the AAC (TPR range from 0.5 to 1) obtained on the independent validation set (1 best, 5 worst). This boxplot shows the median rank along with the quartile ranges for each of the five features.

**Figure S3. Comparison of ranked AAC results.**

Two tables showing a pairwise comparison of the five feature types. Each cell (row=i, column=j) depicts the the p-value obtained by performing a one-sided Wilcoxon rank sum test with as null hypothesis that the median rank of type i is lower than type j, based on the AACs (TPR range from 0.5 to 1) achieved for each of the six experiments. The plot on the left shows individual comparisons, the plot on the right includes comparisons of groups of features. Cell-shading reflects the p-values.

**Figure S4. Comparison of a module-based signature (A) and a gene-based signature (B).**

The module-based signature from the Inter1 experiment contains 55 modules, and the gene-based signature contains 21 genes (Table 1). For both signatures an enrichment score for their overlap with the collection of 2682 gene sets was calculated based on the hypergeometric distribution. This resulted in a total of 319 gene sets that were enriched in at least one module or in the gene-based signature ($p<0.05$ after Bonferroni correction). Several modules turned out to have a similar pattern of enrichment across the gene sets. Additionally, gene sets that relate to a common theme turned out to have a similar enrichment pattern across the modules. Therefore, we clustered the matrix of p-values in both dimensions (2-dimensional, hierarchical clustering, complete linkage, Euclidean distance). The dendrograms at the top, and to the left indicate the clustering, where we chose to group either dimension into seven distinct groups. The labels on the right indicate the individual gene set labels, and the label on the bottom indicates the groups of modules formed along with the number of modules in each group in brackets. The main table shows the median p-value for the enrichment of each of the seven clusters of modules, across these seven groups of gene sets. Similarly, the table on the right shows the median p-values for the gene signature. Shading of the cells reflects the p-values.
