# Supplementary material for: Module-Based Outcome Prediction Using Breast Cancer Compendia
Source: PLoS One. 2007 Oct 17;2(10):e1047. doi: 10.1371/journal.pone.0001047 (PMC2002511; doi:10.1371/journal.pone.0001047)
Supplement: Table S1 — (0.03 MB DOC) [file pone.0001047.s007.doc]

**Table S1. AUC performances across the six experiments**

Table indicating the AUCs for each of the features in the six experiments (AUC column). Within each of the experiments the features were ranked according to their performance. The ranking (R column) ranges from best (rank 1) to worst (rank 5).

| Feature | Intra1 |  | Cross1 |  | Inter1 |  | Intra2 |  | Cross2 |  | Inter2 | |
| --- | --- | --- | --- | --- | --- | --- | --- | --- | --- | --- | --- | --- |
|  | R | AUC | R | AUC | R | AUC | R | AUC | R | AUC | R | AUC |
| BC | 1 | 0.691 | 1 | 0.655 | 3 | 0.613 | 1 | 0.671 | 1 | 0.672 | 2 | 0.648 |
| BCC | 2 | 0.673 | 4 | 0.637 | 3 | 0.612 | 2 | 0.629 | 3 | 0.660 | 2 | 0.649 |
| HCC | 4 | 0.633 | 5 | 0.628 | 2 | 0.620 | 3 | 0.625 | 2 | 0.661 | 5 | 0.575 |
| S456 | 5 | 0.626 | 3 | 0.646 | 1 | 0.633 | 4 | 0.527 | 4 | 0.556 | 4 | 0.605 |
| Genes | 3 | 0.671 | 2 | 0.648 | 5 | 0.591 | 5 | 0.498 | 5 | 0.517 | 1 | 0.709 |
