# Supplementary material for: Module-Based Outcome Prediction Using Breast Cancer Compendia
Source: PLoS One. 2007 Oct 17;2(10):e1047. doi: 10.1371/journal.pone.0001047 (PMC2002511; doi:10.1371/journal.pone.0001047)
Supplement: Table S2 — (0.03 MB DOC) [file pone.0001047.s008.doc]

**Table S2. AAC performances across the six experiments**

Table indicating the AACs (Area Above the Curve) for each of the features in the six experiments (AAC column). AAC values were obtained by integrating over the TPR interval from 0.5 to 1. Within each of the experiments the features were ranked according to their performance. The ranking (R column) ranges from best (rank 1) to worst (rank 5).

| Feature | Intra1 |  | Cross1 |  | Inter1 |  | Intra2 |  | Cross2 |  | Inter2 | |
| --- | --- | --- | --- | --- | --- | --- | --- | --- | --- | --- | --- | --- |
|  | R | AAC | R | AAC | R | AAC | R | AAC | R | AAC | R | AAC |
| BC | 1 | 0.235 | 1 | 0.263 | 1 | 0.294 | 1 | 0.263 | 2 | 0.274 | 2 | 0.282 |
| BCC | 2 | 0.256 | 3 | 0.276 | 2 | 0.295 | 2 | 0.285 | 2 | 0.275 | 3 | 0.283 |
| HCC | 4 | 0.274 | 5 | 0.289 | 4 | 0.297 | 3 | 0.294 | 1 | 0.267 | 5 | 0.317 |
| S456 | 5 | 0.277 | 4 | 0.277 | 1 | 0.294 | 4 | 0.349 | 4 | 0.322 | 4 | 0.312 |
| Genes | 3 | 0.265 | 2 | 0.274 | 5 | 0.305 | 5 | 0.396 | 5 | 0.377 | 1 | 0.221 |
